# Efficacy of Total Tibialis Anterior Tendon Transfer using Button Anchor in Management of Residual Dynamic Supination in Congenital Talipes Equino Varus

**DOI:** 10.5704/MOJ.2603.006

**Published:** 2026-03

**Authors:** A Ajmera, M Solanki, A Pal, M Kumar, U Tiwari

**Affiliations:** Department of Orthopaedics, Mahatma Gandhi Memorial (MGM) Medical College, Indore, India

**Keywords:** congenital talipes equino-varus (CTEV), dynamic supination, tibialis anterior tendon transfer (TATT), disease specific instrument (DSI), foot posture index (FPI)

## Abstract

**Introduction:**

Congenital talipes equino-varus (CTEV) is amongst one of the most common paediatric foot deformities. Ponseti’s method is the standard way of treatment, however, some patients are left with residual or partially corrected deformities. Dynamic supination is one amongst them, where the foot supinates in swing phase of the gait cycle. It is due to a strong tibialis anterior and its weak antagonist.

**Materials and methods:**

We undertook a prospective interventional study in thirty patients of CTEV with residual dynamic supination deformity and treated them with tibialis anterior tendon transfer (TATT) using a button anchor. Minimum follow-up was six months after the surgery. Functional, subjective and objective evaluation was done using foot posture index (FPI), disease specific instrument (DSI) for clubfoot, clinician satisfaction grading and videotaped functional gait analysis. Statistical analysis was done using paired ‘t’ test and calculating p values.

**Results:**

We achieved good to excellent results in 93.3% of our patients and fair in 6.66%. None of our patients had poor results. Mean FPI improved from -1.93 to +0.3, DSI values also showed a significant reduction from 18.17 +/- 1.09 to 13.37 +/- 1.54 after surgery. A total of 90% had satisfactory gait post-surgery at 6 months follow-up.

**Conclusion:**

Tibialis anterior tendon transfer using a button anchor is effective in treatment of residual dynamic supination deformity.

## INTRODUCTION

Congenital talipes equinovarus (CTEV), widely known as clubfoot, represents one of the most common congenital musculoskeletal anomalies, affecting 1.18 in every 1,000 live births globally^1^. Characterised by a complex foot deformity that includes forefoot adduction, midfoot supination, hindfoot Varus, and ankle equinus. The ponseti’s method, characterised by serial casting followed by achilles tenotomy, has been universally acknowledged for its effectiveness in achieving initial correction of clubfoot deformities^2^.

However, despite the widespread success of the ponseti’s method, a subset of patients develops residual deformities, one of the most challenging being dynamic supination during gait, where during swing phase, foot supinates revealing the residual deformity. It is caused due to strong tibialis anterior and weak antagonists, mainly peroneal and tibialis posterior muscles, which necessitates further intervention^3^.

Tibialis anterior tendon transfer (TATT) has been proposed as an effective surgical intervention for correcting residual dynamic supination by rebalancing the forces acting on the foot^3,4^. The technique was perfected by Ponseti in 1963 and has been around since then. This study aimed to evaluate the effectiveness, complications and patient satisfaction associated with this procedure in present times, when treating patients with residual dynamic supination in children who have been previously treated with ponseti’s method for CTEV correction.

In this study, the whole tibialis anterior tendon was transferred to lateral cuneiform using two incision technique and was anchored using a button on the plantar aspect of foot.

## MATERIALS AND METHODS

This prospective and interventional study was conducted at the Department of Orthopaedics and Traumatology, MGM Medical College and associated Maharaja Yeshwantrao Hospital, Indore, Madhya Pradesh, India in 30ft between January 2020 and January 2022 after approval from the institution’s ethics committee. All children coming to the outpatient department with CTEV aged between 2.5 – 5 years with simple clubfoot and residual dynamic supination deformity, post ponseti treatment were included in the study. Children with rigid or secondary clubfoot, atypical clubfoot, unfit for surgery or patients who have undergone any surgery for their deformity other than tendoachillis tenotomy were excluded from the study.

**Fig. 1:**
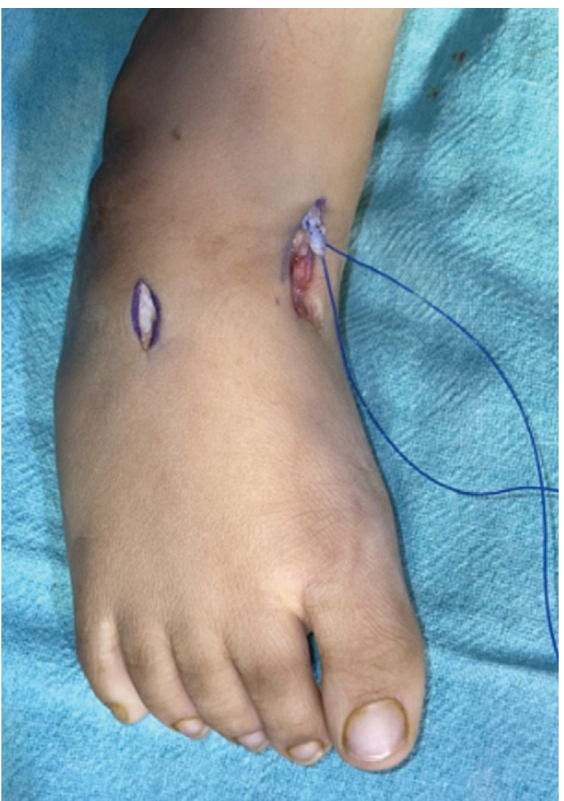
Two-incision technique and the tibialis anterior tendon secured using prolene 1-0.

**Fig. 2:**
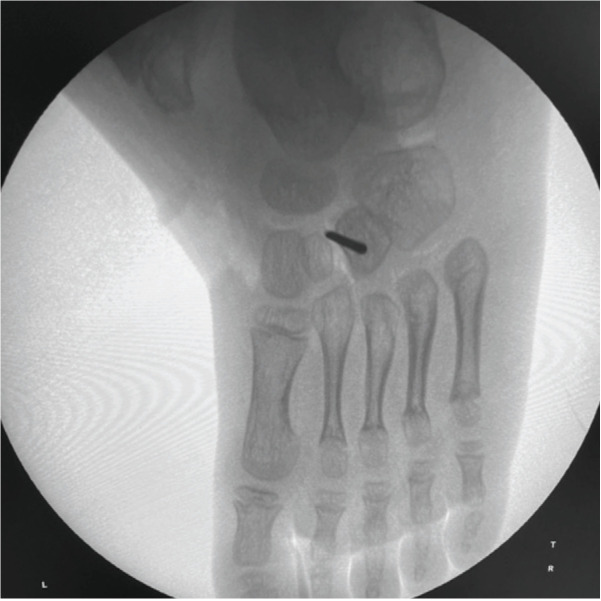
Localisation of lateral cuneiform under fluoroscopy guidance.

**Fig. 3:**
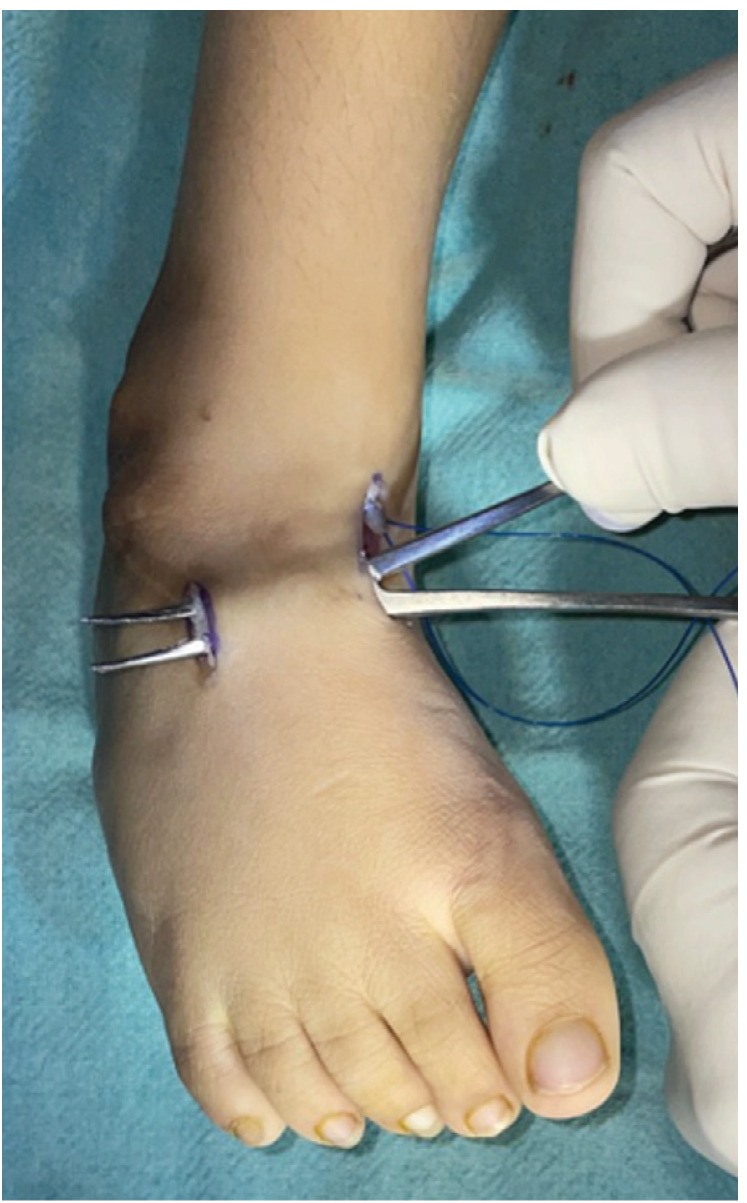
Use of artery forceps to create a subcutaneous space between incisions to reroute tibialis anterior.

**Fig. 4:**
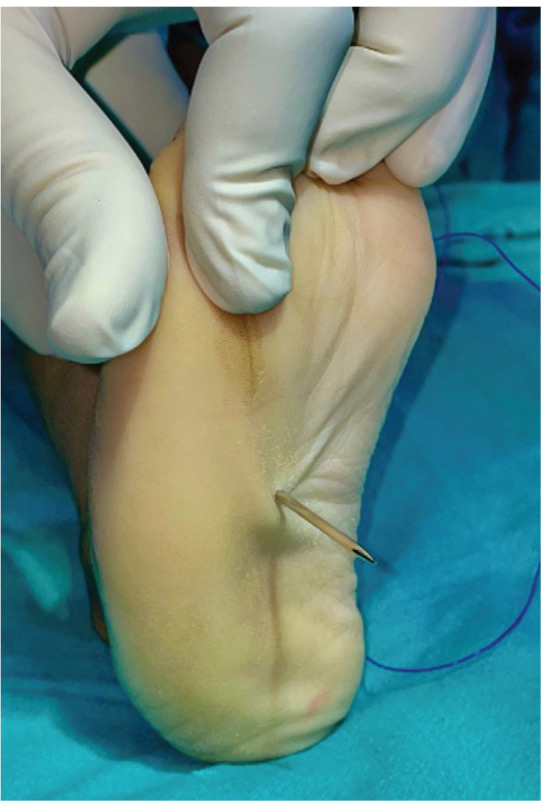
Use of a needle to pass the tendon through the transosseous tunnel created in lateral cuneiform to plantar aspect of foot.

**Fig. 5:**
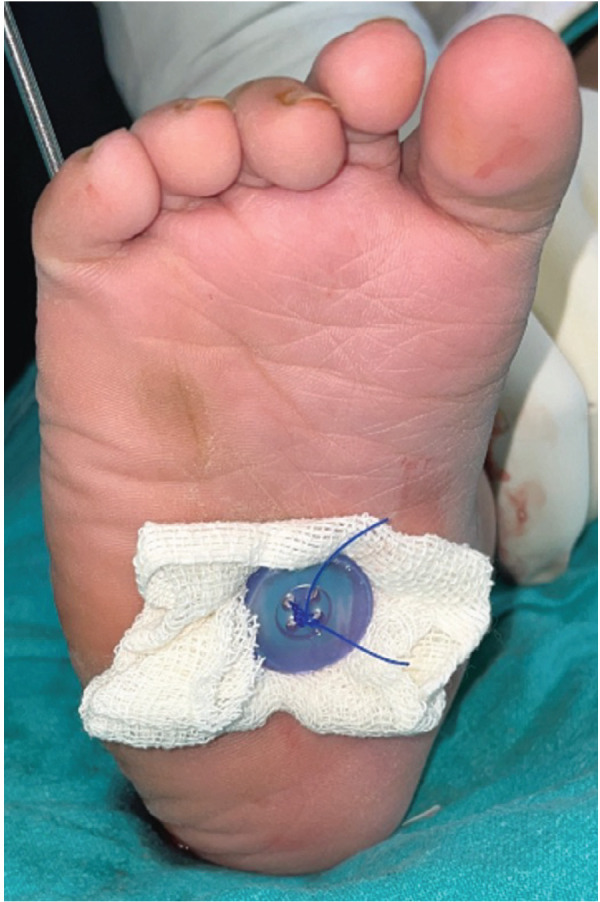
Button anchor to secure the tibialis anterior tendon in the transosseous tunnel in lateral cuneiform.

Tibialis anterior tendon transfer was done using two incision technique (Fig. 1) under Caudal block and sedation, under tourniquet. First incision around 2 – 3cm was made dorsomedially near insertion of tibialis anterior on medial cuneiform. Extensor hallucis and Tibialis anterior tendons were identified by pulling the tendon and watching for great toe and ankle dorsiflexion. Tibialis anterior tendon was freed 1 – 2cm above extensor retinaculum and severed from its insertion along with periosteum from medial cuneiform to gain length and secured using Kraków’s sutures with prolene 1-0 (Fig. 1). Second dorsal incision (2 – 3cm) was taken at the level of lateral cuneiform under fluoroscopy guidance (Fig. 2) and transosseous tunnel was created in lateral cuneiform using k-wires and straight artery forceps. A subcutaneous space was made between the two incisions using a blunt hemostat and tibialis anterior tendon was rerouted through this tunnel from medial incision to its new insertion site on lateral cuneiform in the lateral incision (Fig. 3). It was then passed through a tunnel in lateral cuneiform with help of straight needle (Fig. 4) passed out and anchored on plantar aspect of foot with help of a button (Fig. 5) and prolene sutures in maximum dorsiflexion. Tourniquet was then deflated, and haemostasis was achieved. Wound was closed and above knee cast was applied with knee in 30° flexion. Children were not allowed to walk on the cast and parents were counselled regarding same to prevent them from bearing weight.

Patients were followed-up for minimum of six months after surgery. First follow-up was at three weeks when suture removal was done and below knee cast was given for another three weeks, after which button anchor was removed, eversion exercises were explained, and child was allowed to walk. A foot abduction brace for given to patients for nighttime use for another six weeks. Further follow-ups were done at three months, six months and six monthly thereafter for assessment of foot posture index, videotaped observational gait analysis.

The efficacy of the intervention was evaluated using a combination of functional and subjective tools. Foot posture index (FPI-6)^5^ was calculated where neutral foot is graded as zero, pronated foot is given positive values, and supinated foot is given negative values. Based on these a score range of -12 to +12 was possible, with scores between -12 to -5 being classified as highly supinated, -4 to -1 as supinated, 0 to +5 as neutral, +6 to +9 as pronated and +10 to +12 as highly pronated^6^. Videotaped observational gait analysis^7^ was done, the video was evaluated by three people (two orthopaedic surgeons and one physiotherapist). According to dynamic supination deformity observed, they were graded as: Grade I: no deformity, Grade II: mild, Grade III: moderate and Grade IV: severe deformity observed. Lastly, the parent's satisfaction was assessed utilising a disease specific instrument (DSI)^8,9^ developed by Roye *et al* (Table I). It is a series of 10 questions asked to the parents regarding their satisfaction with the status and appearance of their child’s foot, difficulty in finding fitting shoes, limitations in walking, running, exercising and pain while doing these actions. Each question is graded from 1 to 4, 1 being very satisfied / no limitation in doing the activity and 4 being very dissatisfied / very limited activity. Statistical analysis was done using Paired ‘t’ test, p value <0.05 was taken to be statistically significant.

**Table I:** Disease specific instrument for clubfoot (Roye *et al* - 2001)

**S. No.**	**Questions**	**Response**
1	Are you satisfied with status of the child’s foot?	1-very satisfied, 2-somewhat satisfied, 3-somewhat dissatisfied, 4-very dissatisfied
2	How satisfied are you regarding how the child’s foot looks?	1-very satisfied, 2-somewhat satisfied, 3-somewhat dissatisfied, 4-very dissatisfied
3	How frequently is the child teased regarding how the foot looks?	1-never, 2-sometimes, 3-usually, 4-always
4	How frequently do you have difficulty in finding fitting shoes for your child?	1-never, 2-sometimes, 3-usually, 4-always
5	How frequently does your child have difficulty in finding the shoes that he likes?	1-never, 2-sometimes, 3-usually, 4-always
6	Does your child complain of pain?	1-never, 2-sometimes, 3-usually, 4-always
7	How limited is the child’s ability to walk?	1-not limited, 2-somewhat limited, 3-moderately limited, 4-very limited
8	Does the child have difficulty in running?	1-not limited, 2-somewhat limited, 3-moderately limited, 4-very limited
9	Does the child complain of pain during moderate exercise?	1-never, 2-sometimes, 3-usually, 4-always
10	Does the child complain of pain during heavy exercise?	1-never, 2-sometimes, 3-usually, 4-always

## RESULTS

Study included 24 patients with 30ft having residual dynamic supination deformity after clubfoot treatment. Eighteen patients (18ft) had unilateral clubfoot and 6 had bilateral disease (12 ft). Majority of them were of male sex, male:female ratio was 3.8:1. Mean age of our patients was 3.23 +/- 0.55 years, 62.5% patients were in age group of 2.5 years to 3 years. A total of 23.3% (7 ft) observed history of slippage of casts, moreover, all feet had short and hyperextended first toe which was atypical feature. A total of 26 (86.6%) and 27 (90%) feet had deep plantar crease (plantaris) and swollen, chubby foot, respectively. A total of 23.3% had history of previous treatment with ponseti cast.

As per foot posture index (Table II), out of 30 patients who had supinated feet (score range between -1 to -4) pre-operatively, 27 (p-value <0.05) achieved neutral feet (score range between 0 to +2) post-operatively and 3 still had residual supination in their feet. Comparison of pre-operative and post-operative disease specific instrument (DSI)^8,9^ for clubfoot based on 10 questions asked from parents were as in Table III. Before surgery, mean DSI was 18.17 +/- 1.09, which decreased to 13.37 post-operatively, which was significant (p value of 0.001) showing parent’s satisfaction with clinical outcomes. All our patients were of walking age. Videotaped gait analysis when found to be adequate by 2 out of 3 examiners was labelled as satisfactory. Twenty-seven patients had satisfactory gait; three patients had residual supination during swing phase.

**Table II:** Foot posture index.

**FPI**	**Pre-operative**		**Post-operative**	
	No. of feet	%	No. of feet	%
+2	0	0	03	10%
+1	0	0	06	20%
0	0	0	18	60%
-1.0	03	10%	03	10%
-2.0	26	86.66%	0	0
-3.0	1	3.33%	0	0
-4.0	0	0	0	0
Total	30 (mean= -1.93)	100	30 (Mean= +0.3)	100

**Table III:** Disease specific instrument for club foot.

**DSI**	**Number of Patients**	**Mean ± SD**	**‘T’ value**	**P Value**
Pre-operative	30	18.17 ± 1.09	18.157 Df = 29	0.001*
Post-operative	30	13.37 ± 1.54		

There were four complications in our study, where in one patient had superficial infection over dorsal incisions, which was found during first follow-up at three weeks during suture removal. It was treated with regular dressing and broad-spectrum antibiotics. Three patients had infection on plantar aspect of foot after button removal which improved with dressings in one case and two needed removals of redundant suture under short GA (general anaesthesia) in OT (operation theatre). Of these three patients had residual supination (FPI -1) at the end of six-month follow-up. None of our patients had overcorrection.

## DISCUSSION

Ponseti’s method has been accepted as standard treatment of clubfoot worldwide, however recurrence rate with this method can be from 26-48%^10,11^. One of the common modes of recurrence is residual dynamic supination of the foot where foot supinates as soon as the child lifts his foot off ground during swing phase of gait cycle but appears to be normal when child is bearing weight on the foot. This is postulated to be due to a strong tibialis anterior tendon working against weak peroneii and tibialis posterior muscles^12^. Tibialis anterior tendon transfer has been described in multiple studies to be effective in relapsed clubfoot by restoring balance between eversion and inversion strength of foot while also correcting hind foot varus^11^. Two methods have been described, i.e. a Split Tibialis anterior transfer and a Total Tibialis anterior transfer to lateral cuneiform. There are no significant differences in the final outcomes between the two as per available literature^13^. We therefore did complete transfer of tibialis anterior for correction of residual dynamic supination deformity in clubfeet using button suture anchor and bone tunnel technique.

In our study we had 30ft from 24 patients who had residual dynamic supination after ponseti’s clubfoot treatment with mean age of 3.23 years which was lower than other similar studies conducted by Thompson *et al*^14^ and Lampasi *et al*^15^ who reported their mean ages 4.3 years and 4.8 years, respectively. We had operated on patients when not much improvement was seen in the dynamic supination deformity for six months. Also, in our setup, most of the patients come from far off places from rural areas, hence intervention was done earlier than recommended. Out of 30, significant improvement was seen in 27ft. Mean Foot posture index preoperative was -1.93 which improved to +0.30 (p-value <0.05). Parents satisfaction as calculated by Disease specific instrument for clubfoot was also significantly better post-operatively (p = 0.001), these were also correlated by videotaped functional gait analysis. Thompson *et al*^14^ used their own grading system, which evaluated the restoration of muscle balance and correction of dynamic supination. They were graded as: - good: restoration of muscle balance, fair: partial restoration and poor: no improvement. They reported good results in 87%, fair in 13%. Critchley *et al*^16^ also used similar criteria and reported good results in 72.72%, fair in 9.09% and poor in 18.18% patients, results of which correlate with our study. Using Thompson’s grading criteria, we had 86.6% good results, fair in 13.2% patients and none poor results, which was consistent with other similar studies. Patients were kept in an above knee cast for three weeks, followed by below knee cast for another three weeks. This was found to be a bit challenging as the patients were of walking age group. Parents were counselled thoroughly regarding same, to prevent their child from walking.

However, the follow-up duration of this study was of only six months after the surgery, which is quite less to comment on the long-term efficacy of this procedure of transferring tibialis anterior laterally using a button anchor. Also, this study evaluated only 30ft, in which the surgery was done, thus a larger study with more sample size and longer follow-up duration is required to confirm the findings of this study.

## CONCLUSION

Tibialis anterior tendon transfer using button suture anchor with bone tunnel technique in treatment for residual dynamic supination deformity of clubfoot is very effective and has shown promising results with good to excellent parents as well as surgeon’s satisfaction.

## CONFLICT OF INTEREST

The authors declare no potential conflict of interest.
